# Choosing Wisely in pediatric healthcare: A narrative review

**DOI:** 10.3389/fped.2022.1071088

**Published:** 2023-01-10

**Authors:** Sandra Trapani, Alessandra Montemaggi, Giuseppe Indolfi

**Affiliations:** ^1^ Pediatric Unit, Meyer Children's Hospital IRCCS, Florence, Italy; ^2^ Department of Health Sciences, University of Florence, Florence, Italy; ^3^ Department NEUROFARBA, University of Florence, Florence, Italy

**Keywords:** choosing wisely, children, health care, overuse, overtreatment

## Abstract

**Background:**

It has been estimated that 20% of the tests and therapies currently prescribed in North America are likely unnecessary, add no value, and may even cause harm. The Choosing Wisely (CW) campaign was launched in 2012 in the US and Canada to limit the overuse of medical procedures in adult and pediatric healthcare, to avoid overdiagnosis and overtreatment.

**Methods:**

In this narrative review, we describe the birth and spread of the CW campaign all over the world, with emphasis on CW in pediatric healthcare.

**Results:**

To date, CW has spread to more than 25 countries and 80 organizations, with 700 recommendations published. The awareness of medication overuse also made its way into pediatrics. One year after the launch of the CW campaign, the American Academy of Pediatrics and the pediatric section of the Society of Hospital Medicine provided the first recommendations specifically aimed at pediatricians. Thereafter, many European pediatric societies also became active in the CW campaign and published specific top-5 recommendations, although there is not yet a common set of CW recommendations in Europe.

**Discussion:**

We reviewed the main pediatric CW recommendations in medical and surgical fields and discussed how the recommendations have been produced, published, and disseminated. We also analyzed whether and how the CW recommendations impacted pediatric medical practice. Furthermore, we highlighted the common obstacles in applying CW recommendations, such as pressure from patients and families, diagnostic uncertainty, and worries about legal problems. Finally, we highlighted the necessity to foster the CW culture, develop an implementation plan, and measure the results in terms of overuse decline.

## Introduction

In the last decade, physicians and pediatricians were at a crossroads: on the one hand, the growing availability of sophisticated new tests and therapeutic options, and on the other, the need for safe and quality medicine without wasting excess. It has been estimated that in North America, 20%–30% of prescribed tests and therapies are likely unnecessary, add no value, and may even cause harm (1). The Choosing Wisely® (CW) campaign, launched in 2012 by the American Board of Internal Medicine in the US and then in Canada, was born with the aim to reduce wasteful and unnecessary medical overactivity in adult healthcare. Medical overactivity encompasses overdiagnosis (i.e., when an actual abnormality is discovered but detection of that abnormality does not benefit the patient) (2) and overtreatment (i.e., medical treatments or surgical procedures that are unlikely to improve patient health, while even inflicting unnecessary risks) (3).

**Figure 1:**
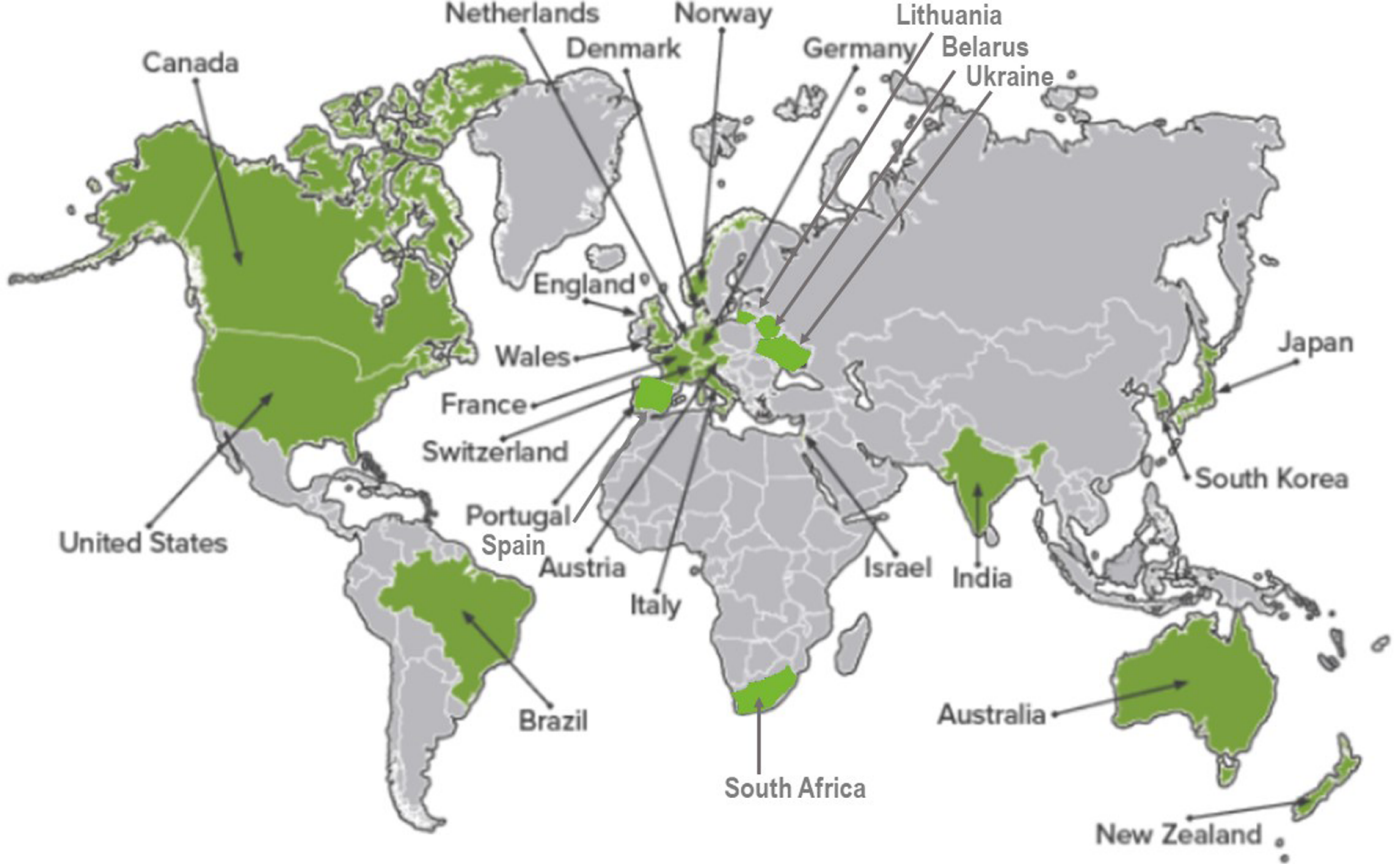
countries that embraced CW campaign.

CW has rapidly spread worldwide through published articles and international and national meetings and, up to now, more than 25 countries on five continents are involved (4). CW campaigns have been founded in the US, Canada, Italy, Australia, Switzerland, the Netherlands, England, Germany, Austria, Japan, New Zealand, Wales, Brazil, Israel, France, and Norway. Other countries, such as South Korea, Denmark, Japan, Singapore, Portugal, Poland, Spain, South Africa, and Saudi Arabia are launching CW campaigns (5). Recently Ukraine, Belarus, and Lithuania joined the movement, too (Figure 1).

CW recommendations are developed by professional scientific societies and aim at identifying commonly used tests and treatments that are not supported by evidence and could expose patients to harm. Since the program's launch, more than 80 organizations joined the CW campaign and generated lists of unnecessary tests and treatments in their specialty. These efforts have produced more than 700 recommendations published worldwide (6). The CW recommendations are not intended to impose strict decisions, but rather, to spur conscious choice about what is an appropriate treatment, considering that each patient's situation is unique. Indeed, the word “routinely” often used inside the recommendations themselves, means that such “wise” suggestions are to be considered in most cases, but they are not strict guidelines. Instead, clinical judgment should remain specific and tailored to each patient based on his/her values and needs. CW campaigns aim to produce changes in the awareness and attitudes of physicians regarding the stewardship of health system resources. However, to be effective in the longer term, these campaigns must change physician behavior, increase patient knowledge of overuse, and ultimately decrease the utilization of unnecessary healthcare services (5). In this narrative review, we aimed to analyze how CW has spread in the pediatric healthcare.

## Narrative review of choosing wisely in pediatric healthcare

### Birth of choosing wisely in pediatrics: US and Canada

Since the launch of the CW campaign in 2012, the awareness of medication overuse and its consequences made its way in pediatrics. A commentary by Schroeder et al. challenged pediatricians to incorporate this knowledge into safety and quality movement (7).

In 2013, the first Pediatric Hospital Medicine focused list of CW recommendations was published. The list was generated through a Delphi process convened by the quality and safety Committee of the Society of Hospital Medicine (SHM) and the American Academy of Pediatrics (AAP). SHM and AAP were the first contributors to provide a few recommendations specifically aimed at pediatricians. They produced the first top-5 list of CW recommendations for pediatric healthcare, which included three suggestions related to bronchiolitis management (avoiding chest x-rays, CXR), bronchodilators, and continuous pulse assessment unless children are on supplemental oxygen), one regarding the unusefulness of systemic corticosteroids in lower respiratory tract infections (LRTI), and the last advising against acid-suppression therapy for gastroesophageal reflux (GER) in infancy (Supplementary Table S1) (8).

Several pediatric societies soon became active in the CW campaign by publishing specific top-5 recommendation lists. Especially in the US, pediatric gastroenterologists, pulmonologists, hematologists, rheumatologists, and surgeons, focused on specific tests and treatments which should be avoided. Every year, new CW recommendations from different countries are now published in each area of pediatrics, from neonatology to surgical specialties.

In 2014, the American College of Rheumatology pediatric section was one of the first pediatric subspecialties that summarized a specific top-5 CW list, based on survey data and literature review, providing an opportunity for patients and physicians to discuss the appropriate use of healthcare in their field (9). Two out of 5 recommendations regarded autoantibodies, including anti-nuclear antibodies (ANA) and autoantibody panels. Indeed, autoantibody testing can be useful in the appropriate clinical setting; however, both adult and pediatric rheumatologists caution against indiscriminate autoantibody testing. Initial ANA testing is only reasonable when the patient's history and examination suggest a possible rheumatologic condition. If ANA is negative, further antibody testing is unnecessary. Similarly, rheumatoid factor testing in a child with musculoskeletal pain has little diagnostic utility in the absence of objective signs of rheumatic disease (9).

In 2015, the Canadian Pediatric Society developed a top-5 CW list, after progressive selection starting among seventeen items (Table 1) (10). A few years later, the same society updated its recommendations with advice on gastroesophageal reflux treatment in infants, food allergies, attention deficit disorder, and cough and common cold treatment (11).

**Table 1:** Choosing Wisely list, recommendations by the Canadian paediatric society (11).

1. Do not routinely order nasopharyngeal testing for typical respiratory illnesses unless results are likely to impact management.
2. Do not routinely perform a voiding cystourethrogram in infants after a first febrile urinary tract infection.
3. Do not use continuous pulse oximetry routinely in children hospitalized with acute respiratory illness unless they are on supplemental oxygen.
4. Do not automatically give IVIG as first-line treatment for children with newly diagnosed typical immune thrombocytopenic purpura.
5. Do not use routine radiography in children who present with acute ankle injuries and meet criteria for a low-risk examination.

The American Society of Hematology (ASH) actively participated in the CW campaign; in 2019, it formed a joint task force with the American Society of Pediatric Hematology/Oncology (ASPHO) to evaluate and select items for a pediatric-focused list. ASH and ASPHO identified the most relevant hematologic tests and treatments that healthcare providers and patients should question because they are not supported by evidence, with potential risks of medical or financial costs, and low benefit. Their final recommendations suggested limiting blood tests (avoid routine preoperative hemostatic testing in an otherwise healthy child with no previous history of bleeding, and avoid thrombophilia testing in children with venous access-associated thrombosis and no positive family history), saving blood products transfusions (avoid platelet transfusion in asymptomatic children with a platelet count > 10 × 10^3^/*μ*l if not requiring an invasive procedure, avoid red blood cells transfusion for asymptomatic children with iron deficiency anemia and no active bleeding), and avoiding routine administration of granulocyte colony-stimulating factor for prophylaxis of children with asymptomatic autoimmune neutropenia and no history of recurrent or severe infections (12).

Finally, in 2021, Tchou et al. provided a timely and extensive update to the 2013 CW list for pediatric hospital medicine: they produced and published the last 5 highest-scoring American pediatric recommendations, using a structured approach, with a focus on specific aspects of hospital care such as the length of intravenous antibiotic therapy, the length of stay for febrile infants evaluated for severe bacterial infection, the threshold of phototherapy for neonatal hyperbilirubinemia, the use of narrow-spectrum antibiotic therapy for community-acquired pneumonia, and the appropriateness of intravenous antibiotics in infants with maternal risk factors for sepsis (Supplementary Table S2) (13).

Moreover, every year in the US, a panel of experts publishes updates on pediatric medical overuse of drugs and diagnostics (14–16). In their last report, they reviewed all articles on pediatric medical overuse published in 2020 and identified the ten most impactful ones. Last year's review highlighted four articles addressing overuse in neonates: hypoglycemia treatment thresholds, blood and platelet transfusion thresholds in preterm neonates, and antibiotic prophylaxis for prenatal diagnosis of urinary tract dilation. Furthermore, it included opportunities for higher-value utilization of antibiotics, surgical and other invasive procedures, and follow-up. For instance, the authors stated that there are unclear benefits of adenoidectomy for preschool-aged children with obstructive sleep apnea and that spontaneous pneumothorax in children does not routinely require hospitalization or a chest tube (17). Some reports focused on specific topics to reduce the use of unnecessary diagnostic tests such as imaging procedures. Kakalia et al*.*, in a retrospective study on 81 adolescents with pulmonary tuberculosis, resize the need to perform chest CT, suggesting that promptly obtaining specimens for sputum smear microscopy, molecular testing, and culture for *Mycobacterium tuberculosis* could avoid unnecessary CTs (18). Other suggestions are given by otolaryngologists as not to routinely order a plain film x-ray in evaluating nasal fractures and not to order imaging to distinguish acute bacterial sinusitis from other upper respiratory infections (19).

While most studies on CW have considered pediatric medical diseases, few articles deal with surgical conditions. Firstly, the Canadian Surgeon Pediatric association provided advice on frequent conditions such as umbilical and/or inguinal hernia (i.e., do not routinely order ultrasound), appendicitis (i.e., do not order C-reactive protein or abdomen CT in children with suspected appendicitis), undescended testes (i.e., do not order a routine ultrasound and do not delay referral beyond 6 months of age) (20).

Likewise, five CW Canadian neurosurgery recommendations were produced to support the adequate care of children with common neurosurgical issues such as macrocephaly, plagiocephaly, hydrocephalus, Chiari malformation, and sacrococcygeal midline dimple (Table 2) (21). While these recommendations apply to most children, occasionally, deviation from these recommendations may be clinically reasonable.

**Table 2:** Choosing Wisely Canada: pediatric neurosurgery recommendations (21).

1. Don’t order a CT to initially investigate macrocephaly (order an ultrasound or MRI).
2. Don’t image a midline dimple related to the coccyx in an asymptomatic infant or child.
3. Don’t use CT scans for routine imaging of children with hydrocephalus. Fast sequence nonsedated MRIs or ultrasounds provide adequate information to assess patients without exposing them to radiation or an anesthetic.
4. Don’t recommend helmets for mild to severe positional flattening.
5. Don’t do routine surveillance imaging for incidentally discovered Chiari I malformation.

Recently, McDonough et al*.* developed a list of evidence-based recommendations to help physicians and patients make treatment decisions on common pediatric otolaryngology conditions, such as adenotonsillectomy (19).

Similarly, ultrasound, as a diagnostic imaging modality, is considered unnecessary for cryptorchidism surgical work-up. However, the utilization of pre-referral imaging remains inappropriately high despite CW recommendations and evidence demonstrating its ineffectiveness (22).

Furthermore, the Canadian Paediatric Society has endorsed two CW recommendations focused on polypharmacy: not initiating medication unnecessarily for GER in infants and ADHD in pre-scholar children. Awareness of this issue and its consequences must also grow in pediatrics. Polypharmacy in children is prevalent both in in- and out-patient settings; obviously, most hospitalized children undergo polypharmacy, particularly those admitted to intensive care units. Among outpatients, polypharmacy is frequent in children with neuropsychiatric disorders and complex medical conditions. Several deprescription protocols have been developed; however, no formal guide is available now, especially for “complex” children treated with many drugs prescribed by different specialists (23).

### Pediatric Choosing Wisely in Europe

Between 2013 and 2019 the CW movement in the pediatric field started spreading overseas. Since 2014, numerous European pediatric scientific societies have begun to implement the principles of the CW campaign, and several European countries such as Italy, Norway, the UK, Switzerland, Germany, and Spain have launched their pediatric CW recommendations and initiatives. In the last years, also Ukraine, Lithuania, and Belarus embraced the pediatric CW campaign (24). There is no common CW recommendations list yet in Europe. However, the European Academy of Pediatrics (EAP) encourages member countries to put avoiding overdiagnosis and overtreatment on their agenda. Stordal et al*.* published in 2019 a statement from the EAP on the most common overtesting and overtreatments: antibiotic overuse, overtreatment of bronchiolitis, unusefulness of proton pump inhibitors (PPIs) in infancy, and non-indicated radiological procedures. These are a few examples from the increasing medical literature that challenge pediatricians to reconsider their current practices (25). The first study investigating knowledge and attitude toward medical overactivity in five European countries was published in early 2022. Despite different cultural and economic environments, the patterns and drivers of increased investigations and medicalization are similar: 83% of surveyed pediatricians stated that they experienced over-investigation/overtreatment, and 81% perceived this as a problem; the majority perceived expectations from family and patients as the most relevant driver for overtreatment in their country, followed by use of national guidelines/recommendations, worry for reactions, and reduction of uncertainty (26).

The Norwegian Pediatricians Association proposed its first list of 5 CW recommendations in 2018, and published an update in 2019. Their recommendations address both excesses of treatment (antibiotics, systemic steroids, bronchodilators, antitussives, and mucolytic drugs) and inappropriate diagnostic procedures, such as chest x-rays, esophageal pH-monitoring, and IgE tests (Table 3) (27). In addition, to identify the five most common errors in family pediatrician practice, Swiss Pediatricians produced a pamphlet to be distributed to parents, where the authors explained what to do or not to do during otitis, reflux, bronchiolitis, cough, and gastroenteritis (28). This tool responded well to the need for good communication and dialogue between doctors and patients, a founding principle of CW.

**Table 3:** Norwegian pediatric associations’ recommendations (27).

First Launch 2018
1. Do not routinely use antibiotics in newborns >36–48 h when a bacterial infection is unlikely.
2. Do not routinely use systemic steroids in airway infections (except for moderate/severe pseudo-croup).
3. Avoid routinely chest x-rays and repeated blood samples in bronchiolitis. Oxygen therapy and pulse oximetry should be stopped when the oxygen saturation is >90% in room air.
4. Avoid esophageal pH-monitoring or treatment with antacids or motility agents in infants with regurgitation when growth and development is normal.
5. Avoid taking skin prick tests or IgE panels against food allergens for suspected food allergy without obtaining a thorough history.
Second Launch 2019
1. Avoid routine antibiotic treatment for acute ear infections in children over 1 year of age
2. Avoid taking urine samples from children > 2 months with symptoms and signs of respiratory infection, unless the child is septic, predisposed to UTI or has specific urinary tract symptoms
3. Avoid routinely antitussives or mucolytic drugs in children with cough or breathing difficulties.
4. Avoid taking abdominal x-rays in children with recurrent abdominal pain.
5. Do not routinely give bronchodilators in bronchiolitis.

In the UK, CW was launched in 2016 by the Academy of Medical Royal Colleges as a way to identify tests, treatments and procedures of questionable value, meaning that the appropriateness of their use should be discussed carefully with patients before being carried out. The Royal College of Pediatrics and Child Health (RCPCH) focused its attention on various issues such as chronic constipation, where polyethylene-glycol should be used in preference to lactulose (29), and prolonged seizures for which buccal midazolam or lorazepam should be used, as these are the most effective drugs, in preference to rectal and intravenous diazepam (30).

In Germany, the CW initiatives in infectious diseases include, among their top-5 recommendations on appropriate vaccinations and antibiotics (dose, route, and type), one item specifically tailored toward children about mandatory measles immunization (31).

In Italy, together with the launch of the CW movement in the US, Slow Medicine promoted the campaign “doing more does not mean doing better” based on the same inspiring principles; the three keywords summarizing the Slow Medicine philosophy, “measured” (because it acts with moderation, gradualness, and without waste), “respectful” (as it takes into consideration the patient's values, preferences and orientation), and “equitable” (because it is committed to ensuring appropriate care based on the best evidence for all) are completely aligned with the CW fundamentals (4).

Thereafter, several pediatric societies of various subspecialties proposed specific CW lists, periodically renewed. The Italian Society of Pediatrics (SIP) recently published two lists of new recommendations, the first on SARS-CoV-2 infection in children and adolescents, and the second on procedures for surgical site infection prevention in neonates and children (32). The Italian Society of Pediatric Allergology and Immunology (SIAIP) has made recommendations to optimize the use of healthcare resources (33) (Supplementary Table S3). Similarly, the Italian Panel of the National Guidelines for acute pharyngitis management in children joined the CW initiative through a specific task force and created a final list of 5 items to avoid unnecessary diagnostic procedures and promote the rational use of antibiotics (34). Also, the Italian Society for Pediatric Respiratory Diseases (SIMRI), through an experts-working board, selected significant points, particularly on the unusefulness of CXR in children with suspected non-severe community-acquired pneumonia or asthma, and CT scans without a strict clinical indication (35). Furthermore, the Italian Society of Pediatric Nephrology (SINePe) suggested approaches to select when to perform some usual tests: for instance, urine culture should not be carried out in the absence of typical symptoms of urinary tract infection, bag urine collection should be avoided, and bio-humoral or instrumental exams are not necessary for asymptomatic microhematuria (Supplementary Table S4). All these recommendations with their references, are uploaded to the Choosing-wisely-Italy website and are easily available for physicians, parents, and patients (36).

## Discussion

### Choosing Wisely recommendations' development

Beginning in 2012, health organizations have asked their members to identify tests and procedures commonly used in their field that should be questioned and discussed to help patients receive the best care, supported by evidence, free from harm, and truly necessary. This call to action has resulted in specialty-specific lists of items (37). All the Top-5 lists are created from the work of expert panels and represent specific, evidence-based recommendations. Each society, free to develop its method to create its list, is required to document the process and make it publicly available (38). Most societies used existing quality and safety committees and solicited feedback from their members through surveys or mailings, and many presented their lists to their governing boards for review and approval (8–10, 12, 19). At the end of each top-5 list, how the list was created and a brief selection of key references are reported (6).

Although the CW movement was born in the adult world, it has been rapidly embraced by many pediatric societies. Hence, culturally, pediatricians have always shown high sensitivity to putting the patient at the center of safe and tailored care while not using unnecessary diagnostic investigations and inappropriate therapies. As shown, many pediatric societies have already published several official CW recommendations. Surprisingly, when compared, many lists, even coming from different countries with diverse health systems, show several similarities. This overlap in CW items suggests that some issues (for example, bronchiolitis, asthma, LRTI management, antibiotic overuse, PPI misuse, and incorrect imaging choice) represent a common source of error with over-medicalization around the world.

### Impact of Choosing Wisely

Adoption of CW recommendations in pediatrics has been slow. At present, their knowledge still seems to be partial, as well as their application. The true usefulness of the recommendations for avoiding “low-value” services is not easily measured, and little research has been performed on this aspect, with different results.

On the one hand, Reyes et al*.*, assessing the clinical impact of the first CW recommendations, found a steady reduction in the frequency of overutilization of five “low-value” services described in the CW campaign-Pediatric Hospital Medicine recommendations from 2008 to 2017 in 36 tertiary children's hospitals in the US, before and after the CW recommendations in 2013*.* The authors found that overall decreases in utilization were 36.6% in relievers and 31.5% in CXR for bronchiolitis, 24.1% in acid suppressors for GER, 20.8% in CXR for asthma, and only 2.9% in steroids for LRTI (39).

On the other hand, several studies suggest a low impact of CW recommendations on clinical practice in these areas, even in countries such as the US where they were developed and promoted.

A cross-sectional study on the use of pulse oximetry was carried out in 56 hospitals in the US and Canada (40), where CW recommendation against the use of continuous pulse oximetry monitoring in children with acute respiratory illness who are not on supplementary oxygen were published in 2013 (8). In 3,612 newborns hospitalized with bronchiolitis without receiving supplemental oxygen, the use of pulse oximetry ranged from 2% to 92%, with an average of 46% (40). Although national guidelines and CW recommendations discouraged its use, this study provided evidence for continuous monitoring of pulse oximetry overuse in children with bronchiolitis (39). Furthermore, Quinonez et al*.* had already well-stressed that overuse of technology in terms of pulse oximetry led to overdiagnosis of hypoxemia, creating uncertainty in oxygen supplementation (41).

In another recent cross-sectional analysis by House et al*.* on the prevalence and costs of low-value care in 49 American pediatric hospitals, the authors found these services were costly, but prevalence varied widely (from 1% to 60%) across measured services. Measures on bronchiolitis, community-acquired pneumonia, and asthma that have been targeted for quality improvement initiatives such as CW recommendations, have resulted in the most common and expensive overtreated conditions (42).

### Main obstacles to Choosing Wisely in pediatric healthcare

Reasons for not following CW recommendations are diverse: holding on to old habits, worries about reactions, reducing uncertainty, and patient/family expectations (26). Doing more feels safer because it alleviates uncertainty, particularly when the stakes are high: families might pressure pediatricians to prescribe drugs or perform tests that might not be indicated. In pediatrics, there is even higher anxiety for both parents and frontline clinicians around diagnostic uncertainty of any kind when it comes to children (43).

Other non-scientific factors, such as clinical traditions, pressure from colleagues or the peer-review process, and fear of legal consequences, contribute to the common practice of “defensive medicine,” and the large availability of tests and treatment may be responsible for the habit of excessive use of medical care even by pediatricians (7). In addition, ordering fewer tests is not easier. Indeed, it often requires more vigilance, effort, and a closer follow-up. Furthermore, pediatricians need to improve their focus on qualitative skills, carefully taking the clinical history and physical examination, to avoid unnecessary laboratory and radiological testing.

Besides, it is important to recognize that many tests and treatments for both medical (like asthma, bronchiolitis, and LRTI), and surgical conditions (such as appendicitis) are initially performed in the Emergency Department setting. To properly implement CW practices, increased collaboration between emergency medicine and hospital medicine specialists is crucial to tackling the issue of medical overuse (44, 45). Thus, additional interventions are required for more effective acceptance and dissemination of the CW recommendations for hospitalized children (39).

### Implementation of Choosing Wisely in pediatric healthcare

It is essential to further foster the culture of CW through national and international congresses, intra-hospital meetings, and by involving high users, including general pediatricians, emergency pediatricians, and pediatric sub-specialists. However, although greater communication would be desirable, this is only one step of the complex process “from theory to practice.” The growing interest in CW nudged Feldman L. to create a new section within the Journal of Hospital Medicine called “Choosing Wisely; things we do for no reason (TWDNFR)” (46). TWDNFR is a platform created for provocative discussions of practices that have become ordinary in hospital care but with limited supporting evidence. Although most articles address adult conditions, several pediatric topics are discussed, too. These are meant as a starting place for research and active discussion among hospitalists and patients or families (46).

Despite the enthusiasm to spread CW campaigns worldwide and in different disciplines, there is little research conducted to evaluate the best implementation strategy. Additional interventions are needed for more effective dissemination of the CW recommendations for hospitalized children. Effective acceptance of CW requires a multi-level approach, including extensive education of patients, parents, and clinicians as well as the involvement of the healthcare system.

Cliff et al., through an extensive review, provided a targeted update by analyzing the evidence on interventions that have reduced the use of low-value targeted or motivated services from the CW campaign. They concluded that dissemination of CW guidelines alone produces little success in reducing low-value care; conversely, multiple interventions to implement CW recommendations, particularly those that are clinician-focused and multi-component, have significant effects and give more convincing results (47).

Therefore, after promoting recommendations, it is necessary to develop an implementation plan aimed at putting them into practice, and then measure the results in terms of overuse decline. The main principles of implementation included targeted education/awareness and transparent measurement with audit/feedback. Most importantly, the changes need to be integrated into the ordering process to make it easier for frontline physicians (43). Evaluating whether CW initiatives work is the first point in assessing whether the effort is “worth it.” Determining the impact of CW campaigns is challenging and requires a comprehensive multi-pronged approach, as proposed by Bhatia et al*.* One of the early markers of the impact of CW is the awareness that more is not always better. Unfortunately, the complexity of recommendations means they are not often easy to measure. Surveying physicians is probably the most straightforward, standardizable, and cost-effective way of assessing physicians' awareness and attitudes. Survey data can be used to gauge the magnitude of the problem and the level of awareness guiding the selection of interventions and addressing differences in attitudes, knowledge, and perceptions that would aid in developing specific interventions. Standardized survey tools distributed by organizations can help assess similarities across countries and discover country-specific differences (48).

Reducing the use of medical services that do not improve patients' health is crucial for both efficiency and quality, particularly in high-income countries. As far as cost is concerned, the potential for benefits provided by CW remains to be demonstrated and may ultimately prove to be limited, and a few studies address the issue. In fact, over time, CW campaigns focused more on high-quality care and no harm than on costs (49, 50).

It is worth observing that CW campaigns mostly spread in high-income countries compared to low and middle-income countries. Several factors have been described as barriers to implementing recommendations in low and middle-income countries, including lack of awareness, limited acceptability, and a lack of trust between patients and physicians. However, CW cancer care initiatives have started being promoted in India and Africa (51).

The CW campaign focuses on professional values and patient-physician conversations. Indeed, effective communication between patients and healthcare providers is an essential part of good healthcare. Patients should be given clear and adequate explanations of their condition, as well as information regarding recommended tests, treatment options, and expected results in order to achieve a conscious choice. The CW movement emphasizes the need to build strong dialogues between doctors and patients while providing strategies for physicians to build trust and address patient attitudes and beliefs that more care is not always better care. Communication is key to ensuring that therapeutic choices are shared and understood by the patient; in the same way, physicians should understand the real needs of patients and families.

### Conclusions

In conclusion, the CW campaign will continue to support efforts to engage physicians and patients with their families in these important conversations and implement the recommendations in practice, in addition to the development or update of new specialty society lists of procedures or tests to question nudging pediatrics providers being more selective in the diagnostics and management of common clinical problems. With this review, we aimed to provide a summary and overview of the CW program in pediatric healthcare. In our opinion, as many high-income countries share similar issues regarding medical overuse, the CW culture found fruitful ground to disseminate rapidly from the US and Canada to the rest of the world. However, additional multi-level interventions are needed for the more effective dissemination and application of the CW recommendations in pediatric healthcare.
