# Corrigendum: Choosing wisely in pediatric healthcare: a narrative review

**DOI:** 10.3389/fped.2024.1369648

**Published:** 2024-02-13

**Authors:** Sandra Trapani, Alessandra Montemaggi, Giuseppe Indolfi

**Affiliations:** ^1^ Pediatric Unit, Meyer Children’s Hospital IRCCS, Florence, Italy; ^2^ Department of Health Sciences, University of Florence, Florence, Italy; ^3^ Department NEUROFARBA, University of Florence, Florence, Italy

**Keywords:** choosing, wisely, childhood, appropriateness, diagnosis

A Corrigendum on Choosing wisely in pediatric healthcare: a narrative review By Trapani S, Montemaggi A and Indolfi G. (2023) Front. Pediatr. 10:1071088. doi: 10.3389/fped.2022.1071088

In the published article, there was an error regarding the affiliations for Sandra Trapani. As well as having affiliation “Department of Health Sciences, University of Florence, Meyer Children's Hospital, Florence, Italy”, they should also have “Pediatric Unit, Meyer Children's Hospital IRCCS, Florence, Italy”.

The authors apologize for this error and state that this does not change the scientific conclusions of the article in any way. The original article has been updated.

