# Supplementary material for: Choosing Wisely in pediatric healthcare: A narrative review
Source: Front Pediatr. 2023 Jan 10;10:1071088. doi: 10.3389/fped.2022.1071088 (PMC9871764; doi:10.3389/fped.2022.1071088)
Supplement: Supplementary file 1 [file Table1.docx]

| **Supplement Material**  **Table 1:** Top five Pediatric Hospital Medicine recommendations by Quinonez *et al*. (8) |
| --- |
| 1. Do not order chest radiographs in children with asthma or bronchiolitis. |
| 1. Do not use bronchodilators in children with bronchiolitis |
| 1. Do not use systemic corticosteroids in children under 2 years with a lower respiratory tract infection |
| 1. Do not treat gastroesophageal reflux in infants routinely with acid suppression therapy |
| 1. Do not use continuous pulse oximetry routinely in children with acute respiratory illness unless they are on supplemental oxygen |

| **Table 2:** Top final 5 CW recommendations by Tchou *et al*. (13) |
| --- |
| 1. Do not prescribe IV antibiotics for predetermined durations for patients hospitalized with infections such as pyelonephritis, osteomyelitis, and complicated pneumonia. Consider early transition to oral antibiotics. |
| 1. Do not continue hospitalization in well-appearing febrile infants once results of bacterial cultures (blood, cerebrospinal, and/or urine) have been confirmed negative for 24–36 h if adequate outpatient follow-up can be ensured |
| 1. Do not initiate phototherapy in term or late preterm well-appearing infants with neonatal hyperbilirubinemia if their bilirubin levels are below levels at which the AAP guidelines recommend treatment. |
| 1. Do not use broad-spectrum antibiotics, such as ceftriaxone, for children hospitalized with uncomplicated CAP. Use narrow-spectrum antibiotics, such as penicillin, ampicillin, or amoxicillin |
| 1. Do not start IV antibiotic therapy on well-appearing newborn infants with isolated risk factors for sepsis, such as maternal chorioamnionitis, prolonged rupture of membranes, or untreated group B streptococcal colonization. Use clinical tools, such as an evidence-based sepsis-risk calculator, to guide management |

| **Table 3:** Five recommendations of the Italian Society of Pediatric Allergy and Immunology – SIAIP- by Bernardini R *et al.* (33) |
| --- |
| 1. Avoid contraindicating routinely vaccination in case of allergies |
| 1. Avoid performing routinely allergy testing in children with acute urticaria |
| 1. Avoid prescribing mucolytics in children with bronchial asthma |
| 1. Avoid prescribing routinely immunological tests in children with recurrent respiratory infections |
| 1. Avoid ruling out a food from the diet only for the positivity of skin prick tests and/or specific serum IgE |

| **Table 4:** Five recommendations of the Italian Society of Pediatric Nephrology - SINePe (36) |
| --- |
| 1. Urine culture should not be carried out either routinely or in the absence of the typical symptoms of a urinary tract infection; bag urine collection should be avoided |
| 1. In the case of low-grade or asymptomatic proteinuria, it is not necessary to perform blood tests or complex instrumental exams. In these cases, medical history, physical exam and urine tests (two separate tests performed at least a week apart) are sufficient. The appropriate diagnostic approach for proteinuria should be followed, if proteinuria persists |
| 1. The indiscriminate use of albumin in children with a first episode of nephrotic syndrome is not recommended |
| 1. When asymptomatic microhematuria is detected in a random urine sample, bio-humoral or instrumental exams are not necessary |
| 1. Children under 6 years of age with primary monosymptomatic enuresis do not need to be seen by a specialist, undergo laboratory (except for urinary dipstick) or instrumental testing or start pharmacological treatment |
